# Is cellular energy monitoring more responsive to hypoxia than pulse oximetry?

**DOI:** 10.1007/s11325-020-02104-2

**Published:** 2020-05-26

**Authors:** Guy M. Hatch, Liza Ashbrook, Aric A. Prather, Andrew D. Krystal

**Affiliations:** 1 Reveal Biosensors, Inc, Logan, UT USA; 2 University of California San Francisco, San Francisco, CA USA

**Keywords:** Cellular hypoxia, Sleep disordered breathing, Primary snoring, Cellular energy monitor, Cellular hyperoxia

## Abstract

**Purpose:**

Pulse oximetry is the current standard for detecting drops in arterial blood oxygen saturation (SpO_2_) associated with obstructive sleep apnea and hypopnea events in polysomnographic (PSG) testing. However, cellular energy monitoring (CE monitoring), a measure related to cellular hypoxia in the skin, is likely to be more responsive to inadequate breathing during sleep because during hypoxic challenge, such as occurs during apneic events, regulatory mechanisms restrict blood flow to the skin to preferentially maintain SpO_2_ for more vital organs. We carried out initial proof of concept testing to determine if CE monitoring has promise for being more responsive to hypoxic challenge occurring during sleep-disordered breathing (SDB) than pulse oximetry.

**Methods:**

We assessed both CE monitoring and pulse oximetry in a series of conditions which affect oxygen supply: (1) breathing nitrogen or 100% oxygen, (2) physical exertion, and (3) studying a night of sleep in an individual known to be a loud snorer. We also present the results of a preliminary study comparing CE monitoring to pulse oximetry in eight individuals undergoing standard clinical overnight polysomnography for suspected SDB.

**Results:**

CE monitoring is responsive to changes in cellular oxygen supply to the skin and detects hypoxia during SDB events that is not detected by pulse oximetry.

**Conclusion:**

CE monitoring is a promising tool for identifying pathology at the mild end of the SDB spectrum.

## Introduction

The presence of pathology occurring with sleep-disordered breathing (SDB) is currently identified during polysomnography (PSG) by the presence of a drop in arterial blood oxygen saturation (SpO_2_), as measured with pulse oximetry, or by electroencephalographic (EEG) evidence of sleep disturbance [1–4]. These findings are used as the basis for the widely employed definitions for apneas, hypopneas, and respiratory-related arousals (RERAs), which form the basis of the diagnosis of SDB and determine whether to institute therapy [1–4]. Those events without a drop in SpO_2_ (hypopneas associated only with arousals and RERAs) are detectable only by a decrease in nasal pressure and an arousal but are nonetheless believed to represent challenges to adequate respiration that are pathological because, according to an American Academy of Sleep Medicine Position Statement, they “cause significant, and potentially dangerous, sleep apnea symptoms.” [5]. It is for this reason that these events are included in the definition of SDB in the International Classification of Sleep Disorders, the primary diagnostic manual for sleep disorders [1], are included among the types of SDB events in the American Academy of Sleep Medicine’s Scoring Manual, the standard manual for scoring sleep studies [3, 4], and are a standard part of scoring polysomnographic data for clinical assessment of SDB [5].

Of note, SDB events at the mild end of the spectrum, referred to as “primary snoring” neither cause drops in SpO_2_ nor disturb sleep and, as a result, are not considered pathological [1]. Although there is no proximal measure of pathology occurring during the sleep study (i.e., drop in SpO_2_ or disturbance of sleep), there is evidence suggesting that primary snoring is associated with cardiometabolic disease [5–8].

### Pulse oximetry vs. cellular energy monitoring

Pulse oximetry, invented in 1972, is the product of the search to find a continuous, non-invasive alternative to periodic arterial blood gas testing for tracking the oxygen content of blood. It is now an indispensable tool in medical practice, and is the standard for measuring oxygenation during sleep in PSG. Pulse oximeters measure the light intensity at the peak and trough of photoplethysmogram (PPG) waveforms at red and infrared wavelengths of light after the light has passed through tissue, such as a fingertip. The less than 2% of full scale (FS) pulsatile waveforms (AC) are the portions of the light signals that are most affected by the oxygen saturation of hemoglobin in the arterioles within the light path. Ratio, logarithmic, and fast Fourier transform (FFT) calculations are currently used to produce SpO_2_ percentage output data from these AC measurements. However, these calculation methods either do not include, or mathematically dispense with, the remaining about 98% FS of the red and infrared light signals that are non-pulsating (DC).

A measure that is more responsive to hypoxic stress than pulse oximetry would have the potential to better determine the extent to which hypoxic stress is occurring during hypopneas associated with arousals, RERAs, and primary snoring. This would allow a better characterization of these events than is currently possible, better elucidating the relationship between hypoxia and adverse outcomes, and, for primary snoring, provide a proximal measure of pathology that could serve as the basis for instituting therapy aimed at mitigating the associated adverse consequences. A new biometric that has promise as a measure of cellular hypoxic stress that is more responsive than pulse oximetry is cellular energy monitoring (CE monitoring). In contrast to pulse oximetry, the CE monitor signals utilize the previously unused 98%, non-pulsating DC components of pulse oximeter signals [9]. While further study is needed to fully define the sources of the CE monitor’s photonic signal responses, the available data suggests that this portion of the signal likely indicates the oxygen supply-dependent dynamic status of energy conversion processes within skin, muscle, and other tissue, mitochondria, as well as blood volume [10, 11].

It is important to note that there are several commercial and medical device sensors that are called “tissue oximeters” by their developers, which claim to measure the oxygen saturation of the blood flowing within tissue. These devices typically use infrared light that can penetrate deeper than the skin and claim to measure the average oxygen saturation of the blood hemoglobin (StO_2_) flowing within the deeper tissues, such as muscle and brain. However, the timed (DC) sampling used by “tissue oximeters” includes confounding variable spectral absorption by the tissue itself, resulting in uncertainty as to what is being reported. Accurate measurement of the molecular oxygen tension, or oxygen content, within tissue, such as with a Clark electrode or by the Lumee® implant and sensor system (Profusa, Inc.) [12], respectively, has also been demonstrated, but these devices do not directly indicate cellular oxygen supply, vs. need and have safety limitations to practical, routine use. Movement of blood cells within capillaries in the skin is also detectable using Doppler-shifted laser light, as an index of blood perfusion in the skin, which is reduced under hypoxic stress by reflex vasoconstriction. This information may be clinically useful as indices of cardiopulmonary health, of the molecular oxygen level in the skin, and of blood flow in the skin. However, the cellular metabolic status relative to the oxygen supply must be assumed. None of these sensors can indicate whether the skin is receiving *less than enough oxygen*, *just the right amount of oxygen*, or *too much oxygen*.

The Reveal CE monitor uses once per second timed samples of tissue-diffused and -absorbed light intensity at unique wavelengths and calculates its Cellular Energy Index (CEi) output data by subtracting the detected infrared intensity value from the detected red intensity value. This provides a continuous numeric scale, with a central, or zero, position at the awake, non-stressed condition, and extends with a negative trend with decreased cellular oxygen supply, and a positive trend with excess cellular oxygen supply. A measure of cellular energy is likely to be more responsive to inadequate breathing than pulse oximetry. This is because, in cases of hypoxic challenge, such as occurs with SDB, regulatory mechanisms restrict blood flow to the skin to preferentially conserve limited oxygen supply for more vital organs.

This article reports the results of initial proof of concept testing to determine if CE monitoring more sensitively reflects hypoxia than pulse oximetry. We used a comparison of CE monitoring with pulse oximetry rather than other accurate measurements such as Clark electrodes because: Pulse oximetry is the current standard used in the application of interest (assessment of sleep disordered breathing). Pulse oximetry, unlike other methods, is likely to be familiar to the readers of this article, and there is evidence that Clark electrodes and related methods are not measuring the same thing as CE monitoring.

We assessed both CE monitoring and pulse oximetry in a series of conditions which are known or suspected to affect oxygen supply: (1) exposing individuals to conditions known to under- or over-supply cellular oxygen, including breathing nitrogen or breathing 100% oxygen; (2) studying an individual during physical exertion; and (3) studying a night of sleep in an individual known to be a loud snorer. We also present the results of a preliminary study comparing CE monitoring to pulse oximetry in eight individuals undergoing overnight polysomnography for suspected SDB.

## Methods

**Fig. 1:**
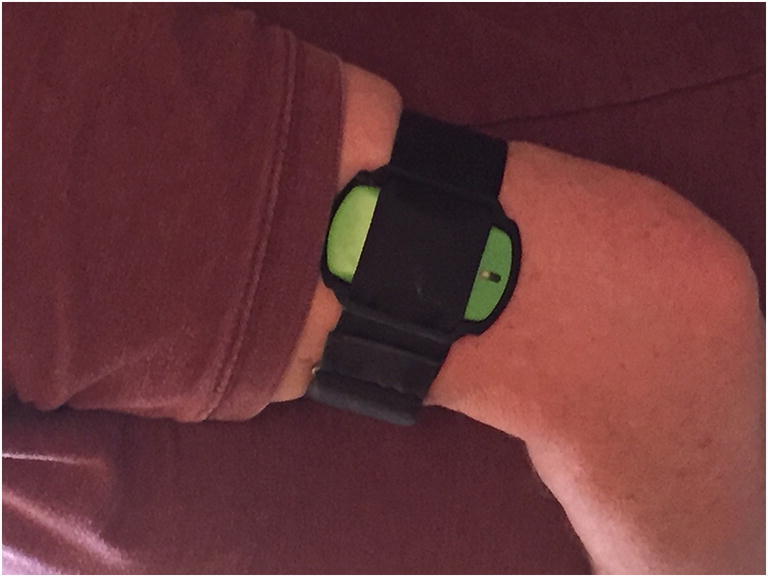
Cellular energy monitor with typical upper arm placement

For all procedures performed, data is collected with the CE monitor sensor placed on the mid-upper arm attached with a watchband-like strap (see Fig. 1). With regard to the Methods for Conditions 1 and 2 below, although standard testing typically employs increases and decreases of oxygen supply for periods of 10 min, we chose to utilize shorter periods for these tests because it highlights the unique capacity of CE monitoring to detect changes in oxygenation within a much shorter period of time. For each of the four conditions described below, SPO_2_ was recorded from a pulse oximeter sensor (signal averaging time of ≤ 3 s at a heart rate of 80 bpm) that was placed on an index finger of the participant prior to and throughout each experiment.

### Methods for initial proof of concept testing

#### Condition 1: inhalation of 100% N_2_

A healthy individual without complaints breathed 100% nitrogen gas for 1 min to create transient hypoxic stress. The CEi derived from the CE monitor sensor and SpO_2_ derived from a standard medical grade pulse oximeter sensor were measured during 1 min of N_2_ inhalation and for 1 min thereafter.

#### Condition 2: inhalation of 100% O_2_

Excess oxygen was delivered to a healthy individual by breathing 100% oxygen for 1 min. The CEi and SpO_2_ (standard medical grade pulse oximeter) were measured during 1 min of 100% O_2_ inhalation and for 1 min thereafter.

#### Condition 3: physical exertion

CEi and SpO_2_ were also obtained as above in a healthy individual during, and for 6 min immediately after, an approximately 14-min session of exercise on a stationary bicycle. The period of exercise included three approximately 2-min intervals of high exertion.

#### Condition 4: sleep in an individual known to be a loud snorer

CE monitoring data was collected during an 8-h PSG in a healthy individual with a clear, self-reported history of consistent loud snoring. The PSG was conducted according to American Academy of Sleep Medicine (AASM) Guidelines [4] and included the following channels: left outer canthus of the eye (LOC); right outer canthus of the eye (ROC); six EEG channels referenced to the contralateral mastoid (F3-M2, F4-M1, C3-M2, C4-M1, O1-M2, and O2-M1); two chin EMG channels; EKG; a sound pressure microphone (snore); nasal pressure (PTAF), oronasal thermocouple (airflow); inductive plethysomnography detection of thoracic and abdominal respiratory effort (chest and abdomen); left and right anterior tibialis EMG (LLeg and RLeg); and pulse oximetry (SpO_2_). The study was scored according to AASM guidelines [3, 4].

### Pilot study methods

We carried out a pilot study where we obtained CE monitoring data simultaneously with PSG data in a convenience sample of eight unselected individuals referred for PSG in the UCSF Sleep Disorders Center as part of their clinical management. The study received approval from the institutional review board of the University of California, San Francisco (Study Number 17-22443), and all participants provided informed consent.

#### Pilot study participants

The participants consisted of eight patients all referred for PSG for suspected obstructive sleep apnea. The sample was a convenience sample consisting of all those individuals who came to the UCSF clinical sleep laboratory for sleep studies and were willing to sign informed consent for participation during the period of recruitment (8/8/2018–10/17/2018). Subject age ranged from 31 to 73 years of age with a mean age of 52.5 years (standard deviation = 16.3 years). Participants were paid $50 for study participation. The subject gender ratio, seven men and one woman, reflects the chance gender distribution in the convenience sample of subjects. None of the participants was undergoing treatment for SDB at the time of their participation.

#### Pilot study procedures

Subjects were approached for participation during the period of the hookup for their clinical PSGs. Those who provided informed consent underwent CE monitoring data collection simultaneously with PSG. The device was placed on the arm of the subjects’ choosing as our assumption was that the choice of arm does not affect device function. As a result, data on the arm the device was attached to were not collected.

The PSG data was obtained and scored according to American Academy of Sleep Medicine (AASM) Guidelines as described above [3, 4]. This included that SPO_2_ was recorded from a pulse oximeter sensor (signal averaging time of ≤ 3 s at a heart rate of 80 bpm) that was placed on an index finger of the participant prior to and throughout each experiment. The Apnea-Hypopnea Index was calculated as the total number of apneas plus the total number of hypopneas that met scoring criteria for these events based on the AASM scoring guidelines divided by the total sleep time [3, 4].

**Fig. 2:**
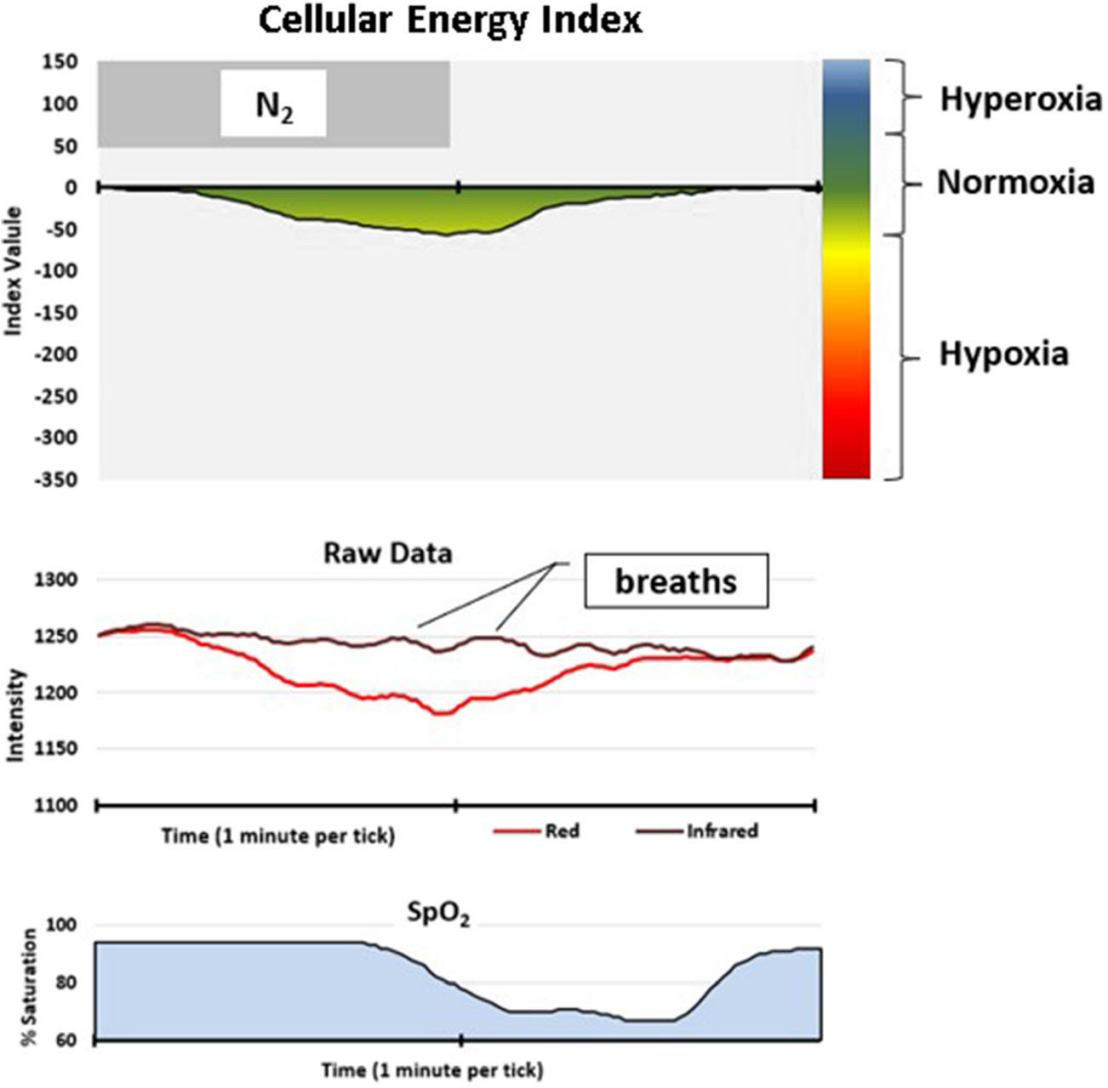
Cellular Energy Index (CEi) recording while breathing nitrogen (N_2_) for 1 min

**Fig. 3:**
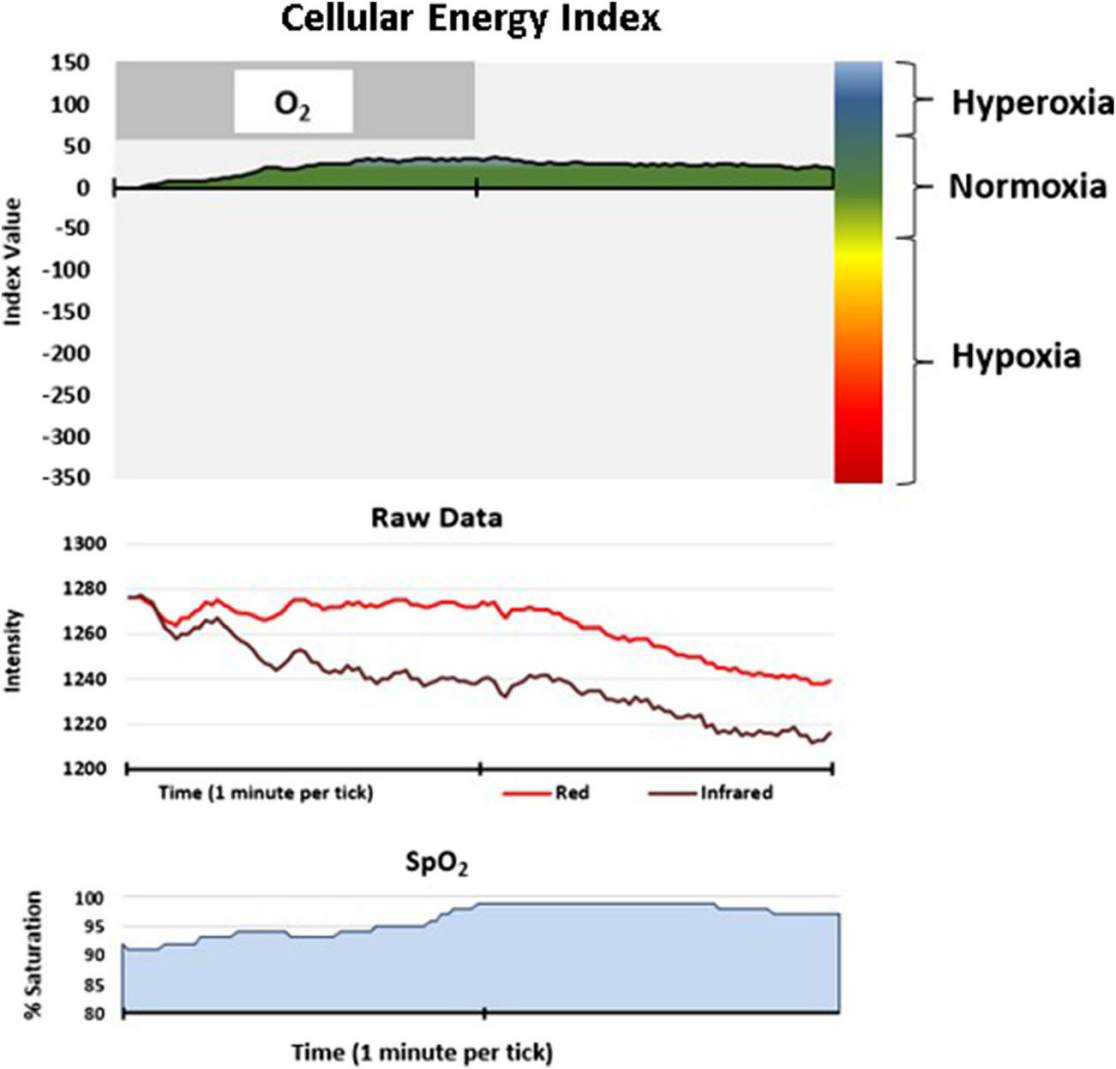
Cellular Energy Index recording while breathing 100% oxygen (O_2_) for 1 min

**Fig. 4:**
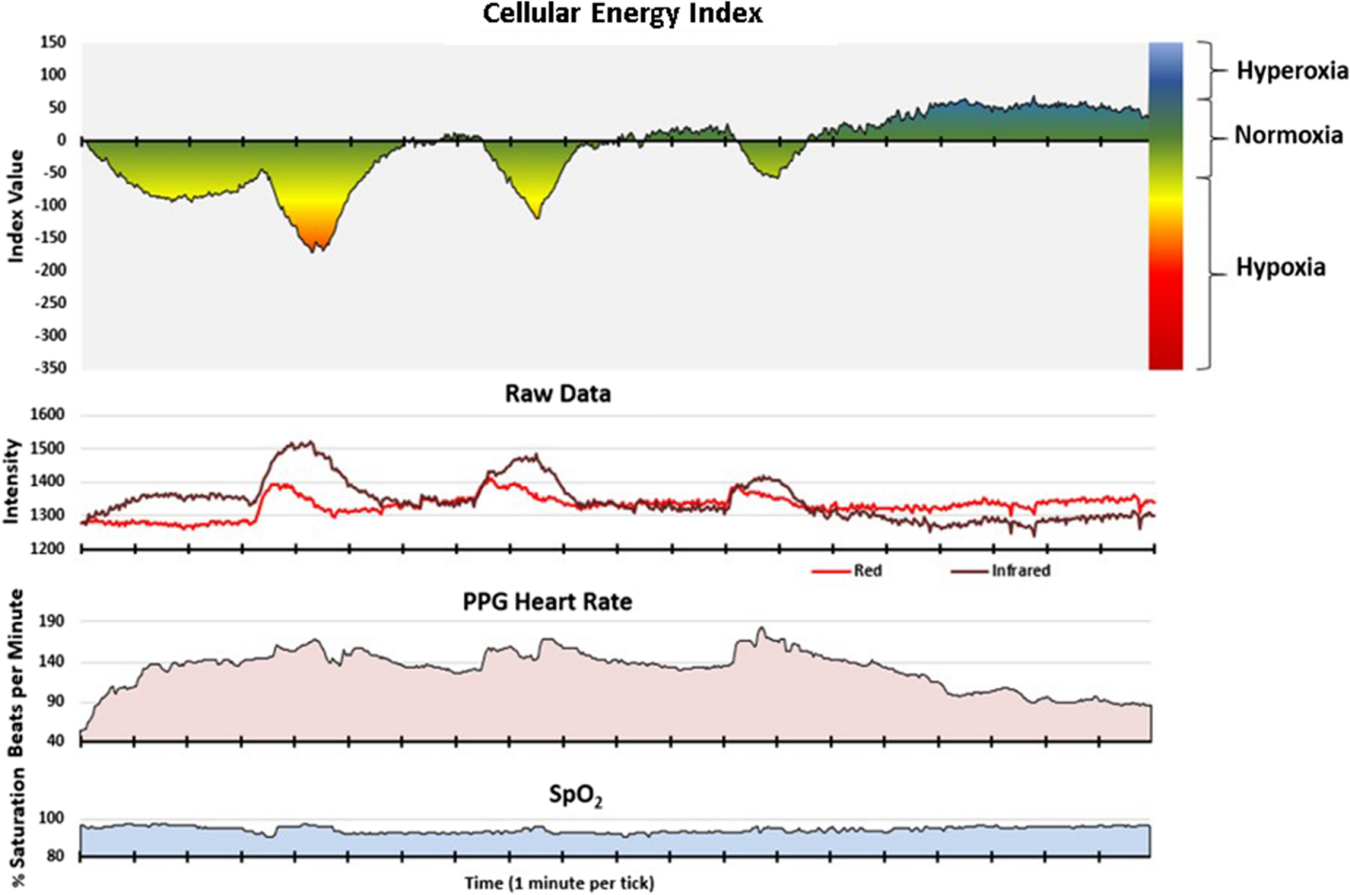
Simultaneous recording during exercise: Cellular Energy Index and the raw data from which it is derived, PPG heart rate, and SpO_2_

**Fig. 5:**
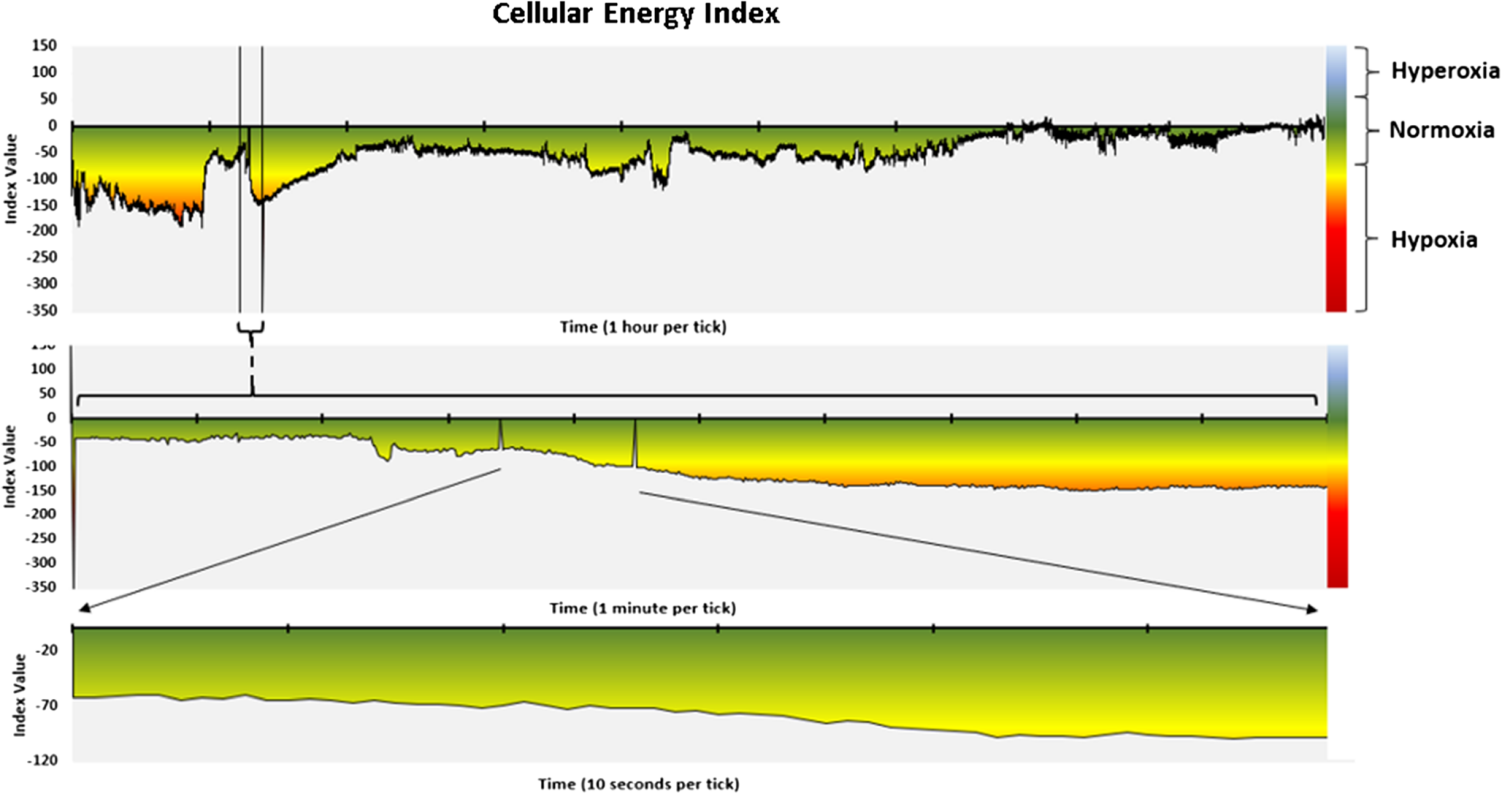
Cellular Energy Index sleep record. These three graphs portray the Cellular Energy Index (CEi) signal throughout a sleep study period. The top graph displays the CEi for the entire 9-h overnight recording. The second graph represents a 10-min period from this recording, selected based on the subject being asleep, snoring, and without change in SpO_2_; but with a declining CEi trend. The third graph is a 1-min sample taken from within this segment, which corresponds with the PSG data appearing in Fig. 6

**Fig. 6:**
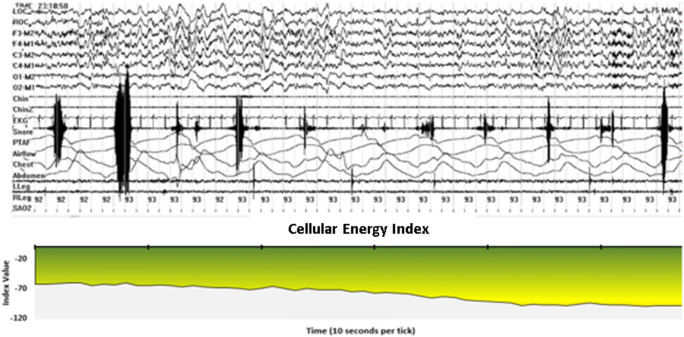
PSG and Cellular Energy Index during primary snoring. This selected one-minute segment depicts data from a standard PSG consisting of the following channels: left outer canthus of the eye (LOC); right outer canthus of the eye (ROC); six EEG channels referenced to the contralateral mastoid (F3-M2, F4-M1, C3-M2, C4-M1, O1-M2, and O2-M1); two chin EMG channels; EKG; a sound pressure microphone (Snore); nasal pressure (PTAF), oronasal thermocouple (airflow); inductive plethysomnography detection of thoracic and abdominal respiratory effort (chest and abdomen); left and right anterior tibialis EMG (LLeg and RLeg); and pulse oximetry (SAO_2_). The data indicate a decreasing CEi trend during a period that the subject is: asleep; snoring; without pulse oximetry desaturation; without awakening; with only very minimal arousal; and without apnea, hypopnea, or obvious hypoventilation

In addition, we scored events based on the CE monitoring data, which we referred to as “hypoventilation events.” We used a criterion of at least a 10-point drop in CEi to define an event. This was an initial criterion based on qualitative review of the data presented in Figs. 2, 3, 4, 5, and 6 which includes one night of CE monitoring data collected during PSG. Lacking criteria for significance, it was understood that it was necessary to set an initial ballpark estimate for an CEi threshold for events. This was done with the understanding that future work would be necessary to set a threshold for clinically significant events based on quantitative methods aimed at optimizing sensitivity and specificity. We employed an event definition of a 10-point drop in CEi accompanied by a drop in peak nasal pressure of ≥ 30% of pre-event baseline nasal pressure lasting at least 10 s because this is the AASM criteria for defining hypopneas; so use of this definition ensures that events were grounded in changes in breathing established as the respiratory basis for defining clinically significant SDB events [3].

##### Pilot study data analysis

Analysis consisted of tabulating events scored on the basis of CE monitoring and comparing those with events detected by standard methods including apneas, hypopneas, and RERAs. No formal statistical analyses were carried out as they were not necessary to characterize the nature of the findings present and achieve the Proof of Concept aims of this study. An analysis for determining if the data distribution was normal was not carried out as the number of subjects was too small to establish a valid distribution.

## Results

### Results of initial proof of concept testing

#### Condition 1: inhalation of 100% N_2_

Figure 2 illustrates that during, and for approximately 30 s after inhalation of 100% N_2_, the CE monitor sensor detects increased absorption of the red wavelength of sensor light, with slight decrease, or no change, in the absorption of the infrared light. This leads to a drop in the CEi during this period.

We also noted variations in amplitude of the infrared signal which corresponded with breath cycles and offer an analog of the effort of each breath.

#### Condition 2: inhalation of 100% O_2_

The CE monitor sensor detected increased absorption of the sensor’s infrared wavelength, with a slight decrease in the absorption of the red light (see Fig. 3). Both of these photonic signal responses become evident within 5–10 s of the onset of changes in the partial pressure of oxygen (ppO_2_) of the breathing gas. Due to extended averaging to suppress motion artifact, pulse oximeter data response is always delayed and this was reflected in the delay of up to 45 s evident in Figs. 2 and 3. Remarkably, prolonged exposure to excess oxygen results in increased absorbance of the infrared light as manifested in a persistent increase in CEi, indicative of excess cellular oxygen supply. We observed that this photonic response persisted for several hours after the excess oxygen exposure was discontinued. The pulse oximeter data increase stopped at 99% in response to excess oxygen supply, then returned to the previous level within minutes.

#### Condition 3: physical exertion

Figure 4 presents CE monitoring and pulse oximeter data recorded simultaneously during a stationary bicycle interval exercise session. The subject’s SpO_2_ remained above 95% during exercise. During the initial phase of exercise, however, the CE monitor detects what appears to be an immediate decrease in cellular oxygen supply. The CEi also decreased significantly during the successive three intervals of high exertion, with a progressive lessening of the photonic responses with successive intervals of near-equal physical effort. Upon stopping exercise, the cellular oxygen supply becomes excessive, despite the fact that the blood volume in the sensor light path (infrared trace of the raw data) immediately returns to its baseline value range.

#### Condition 4: sleep in an individual known to be a loud snorer

Figures 5 and 6 present data from a single 8-h standard clinical PSG with a loud snorer. Figure 5 depicts the CEi trend for the entire night as well as for a selected 10-min period chosen to illustrate a period where the subject was asleep, snoring, and without change in SpO_2_, but with a declining CEi trend. Figure 5 also includes a 1-min period for which raw PSG data appear in Fig. 6 in which this subject is asleep and snoring loudly, is without evidence of blood oxygen desaturation based on pulse oximetry, and where there is only minimal arousal and no evidence of apnea, hypopnea, or hypoventilation; yet, there is a clear decreasing trend in CEi. This selected segment is only one of several such CEi deviations in this study. There were also several prolonged periods of decreased cellular oxygen supply, which were not associated with decreased SpO_2_.

### Pilot study results

Study results appear in Table 1. The Apnea-Hypopnea Index (AHI) for the eight study subjects ranged from 0 to 16.2 events/h indicating that, by chance, the subjects happened to have a relatively low overall severity of SDB. Five of the eight subjects did not have clinically significant SDB by current criteria (AHI < 5).

**Table 1:** Pilot POC study results

Subj	Apnea Hypopnea Index	Number of apneas without skin desaturation	Number of hypopneas without skin desaturation	Hypoventilation Events Index
1	0	0	0	0.2
2	0	0	0	0
3	0	0	0	0.3
4	0	0	0	0
5	0.2	1	0	52.1
6	11.8	0	0	37.1
7	16.2	0	0	35.2
8	8.4	0	0	30.6

All indexes are events per hour of sleep

**Fig. 7:**
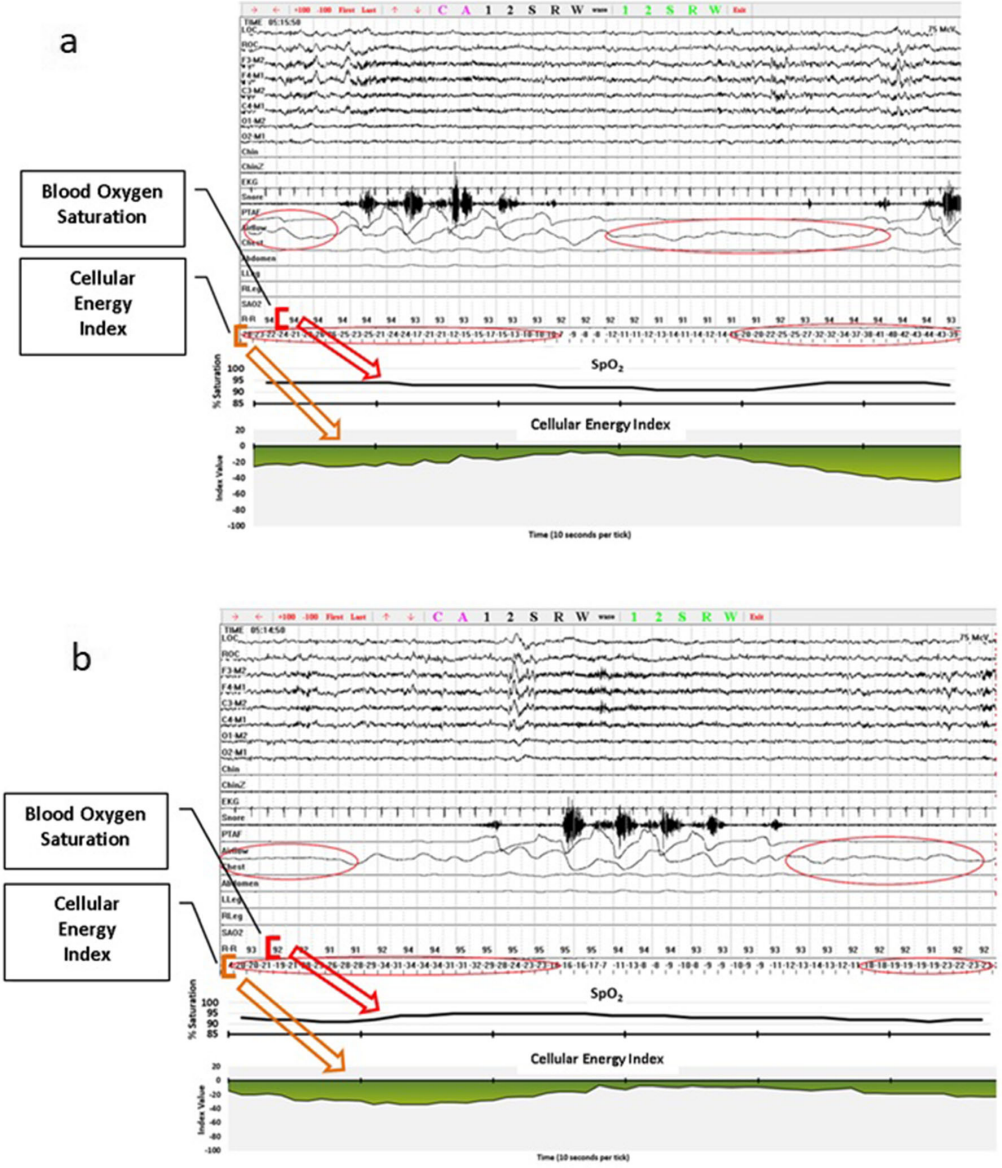
**a** Apnea events accompanied by drops in CEi. **b** Apnea events accompanied by drops in CEi

There was evidence that CE monitoring had a very high level of sensitivity for detecting events defined based on the standard AASM scoring criteria. All apnea and hypopnea events occurring in the eight subjects were accompanied by a drop in CEi (see Fig. 7 for examples) except for one. However, in some subjects, CE monitoring detected a number of events not detected with standard methods. It is not clear if the events detected are clinically significant as this study was not capable of determining clinical significance. It does however establish proof of concept that CE monitoring detects events not detected with pulse oximetry.

**Fig. 8:**
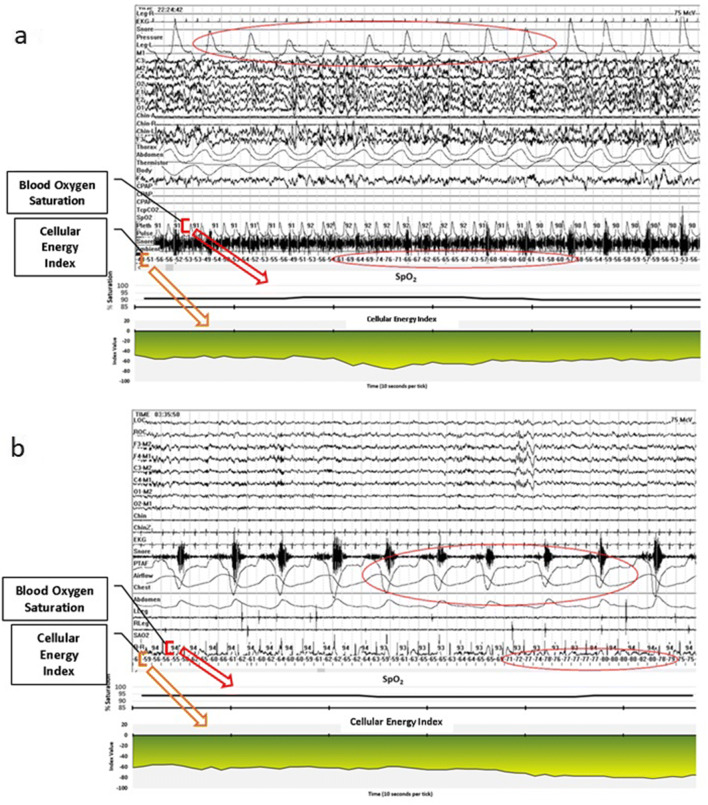
**a** Hypoventilation events (drops in nasal pressure accompanied by 10 point drop in CEi). **b** Hypoventilation events (drops in nasal pressure accompanied by 10 point drop in CEi)

For four subjects who had AHI < 1 the number of hypoventilation events per hour of sleep (Hypoventilation Events Index) was also < 1 per hour. In one subject who had an AHI < 1, the Hypoventilation Events Index was 52.1 (subject 5 in Table 1) indicating a very high rate of events associated with a drop in nasal pressure without an arousal or blood oxygen desaturation, which were accompanied by a drop in CEi (see Fig. 8). For all three subjects who had clinically significant SDB (AHI of 11.8, 16.2, and 8.4), there was a greater rate of hypoventilation events than scorable apneas and hypopneas. For subject 6, the AHI indicated 11.8 events per hour of sleep while there were 37.1 hypoventilation events per hour of sleep. Because all of the apneas and hypopneas were detected with CE monitoring, this indicates that there were 25.3 hypoventilation events per hour not meeting standard criteria for apneas or hypopneas. For subject 7, the AHI was 16.2/h while there were 19 hypoventilation events per hour not meeting apnea or hypopnea criteria. Lastly, subject 8 had an AHI of 8.4/h and 22.2 hypoventilation events per hour not meeting apnea or hypopnea criteria.

There were no adverse events of recording CE monitoring and no complaints regarding use of this device.

## Discussion

The initial proof of concept (POC) testing carried out establishes basic POC that CE monitoring is responsive to changes in cellular oxygen supply to the skin. CE monitoring reflects decreased oxygen supply occurring with breathing 100% nitrogen and exercise and indicates excess oxygen supply occurring with breathing 100% oxygen and during recovery from exercise. Further, initial POC testing suggests that CE monitoring can be recorded during sleep and that it detects drops in oxygen intake in an individual who is snoring but not having scorable apnea or hypopnea events.

Our pilot study provides more convincing evidence for POC that CE monitoring might be more sensitive than pulse oximetry for detecting hypoxic events. CE monitoring sensitively detected events associated with pulse oximetry-detected hypoxemia already established to be associated with pathology (apneas and hypopneas). However, CE monitoring also detected a drop in cellular oxygenation during events established to be associated with pathology where there is no blood oxygen desaturation occurring with pulse oximetry (hypopneas associated with arousals and respiratory related arousals). This suggests the possibility that such events exist on a spectrum of severity with apneas, hypopneas, and RERAs, where the oxygen intake challenge is not sufficient to be detected with pulse oximetry-based blood oxygen measurement but can be detected with CE monitoring. The greater sensitivity of CE monitoring for these relatively milder oxygen intake challenges is consistent with well-known reflex responses that help maintain blood pressure and minimize decreases in oxygen saturation of the arterial blood supplying the brain and other vital organs at the expense of decreased oxygen supply to the skin [13].

In addition to potentially detecting pathology associated with snoring, the CE monitor could increase the sensitivity of home sleep testing. Currently arousal-based hypopneas and RERAS are formally detected during attended polysomnography that includes EEG to provide sleep staging and detect arousals. These events are not picked up on home sleep testing which has become the majority of clinically performed sleep studies to assess for sleep apnea in many regions. If a home monitor could detect these events, it would improve the current home sleep test, which can have a sensitivity of only 80%, and allow for accurate diagnosis and treatment for a greater number of patients [14].

Whether all events picked up by CE monitoring are pathological remains to be determined. However, a reason to think they might be pathological is that such events are accompanied by a decrease in nasal pressure, which is already established as the respiratory basis for defining clinically significant events—hypopneas.

The findings also suggest the possibility that decreased cellular oxygen supply in the skin, as detected by CE monitoring, might be occurring at times in conjunction with snoring even in the absence of decreases in nasal pressure, airflow, or sleep disturbance. This was not directly systematically assessed in this study but should be pursued in future work aimed at determining if CE monitoring could serve as a proximal measure of snoring-induced pathology.

A favorable feature of CE monitoring noted in this study is that its lack of data processing delay compared with pulse oximetry data allows a closer temporal tracking of alterations in oxygenation. In addition, CE monitoring reflects breath-by-breath breathing effort which could be used to differentiate obstructive vs. central respiratory events.

It should be noted that, by chance, there was a relatively low overall level of SDB severity in our pilot study sample. To better determine the potential utility of CE monitoring for detecting SDB events, it will be important that future studies include individuals with more severe SDB. In addition, future studies would benefit from including a greater number of participants as well as a balance of women and men. Future studies designed to optimally determine thresholds for detection of SDB events using CE monitoring will also be needed to address a limitation of the current work, that the CE monitoring threshold for identifying events could not be determined via a systematic analysis. It was first necessary to establish proof of concept that CE monitoring was responsive to changes in oxygenation in general and occurring during sleep before optimizing thresholds for detecting events associated with hypoxic stress.

## Abbreviations

AC: Alternating current
APAP: Auto-titrating continuous positive airway pressure
CEi: Cellular Energy Index
CE: monitor Cellular energy monitor
COPD: Chronic obstructive pulmonary disease
CSA: Central sleep apnea
DC: Direct current
EEG: Electroencephalogram
FFT: Fast Fourier transform
FS: Full scale
O_2_: Oxygen
OSA: Obstructive sleep apnea
POC: Proof of concept
PPG: Photoplethysmogram
PSG: Polysomnogram
SDB: Sleep disordered breathing
SpO2: Arterial blood oxygen saturation
